# X-linked dominant chondrodysplasia punctata with severe phenotype in a female fetus: A case report

**DOI:** 10.1097/MD.0000000000013850

**Published:** 2019-01-04

**Authors:** Yan Liu, Li Wang, Bin Xu, Yike Yang, Dan Shan, Qingqing Wu

**Affiliations:** a Department of Obstetrics; b Department of Ultrasound; c Department of Radiology, Beijing Obstetrics and Gynecology Hospital, Capital Medical University, Beijing 100026, China.

**Keywords:** CDPX2, 8-dehydrocholesterol, EBP, X-chromosome inactivation

## Abstract

**Rationale:**

X-linked dominant chondrodysplasia punctata type 2 (CDPX2) is a condition involving facial, skin, and skeletal dysplasia as a result of a mutation in emopamil binding protein (*EBP*). It usually presents with mild symptoms in female patients but is fatal in male patients.

**Patient concerns:**

A fetus was diagnosed with asymmetrical short limbs and a narrow and small thorax by prenatal ultrasound examination at 24+5 weeks gestation. The pregnancy was terminated at 27 weeks of gestation; gross examination, postnatal X-ray and, whole exome analysis were performed to clarify the diagnosis.

**Diagnosis:**

A provisional diagnosis of fatal skeletal dysplasia was given and the definite diagnosis of CDPX2 was based on postnatal X-ray and genetic testing of the aborted fetus.

**Intervention:**

The pregnancy was terminated at 27 weeks’ gestation after a fetal ultrasound indicated a severe abnormal phenotype.

**Outcomes:**

Whole exome analysis of aborted tissue confirmed *EBP* mutation in this case. Unlike most case reports, this female patient presented a severe phenotype that was considered to be related to X-chromosome inactivation.

**Lessons:**

Chondrodysplasia punctata (CDP) should be considered if prenatal ultrasound shows high punctuate echoes at the metaphysis of long bones and asymmetrical short lower limbs. Postnatal X-ray and measurement of sterol levels in the amniotic fluid may aid in the diagnosis of CDP, but the condition can be confirmed with genetic testing of a blood sample or aborted tissue after delivery.

## 1 Introduction

Chondrodysplasia punctata (CDP) is a group of skeletal dysplasias caused by congenital cartilage ossification disorders with different genetic patterns. It is characterized by irregular calcium deposits in the metaphyseal cartilage that form punctate calcification and scattered small calcifications at the metaphysis of long bones. The leading cause of this condition is genetic mutation that leads to chromosomal aberration or congenital enzymatic metabolic disorders. The clinical manifestations, genetic, and etiological classifications are quite complex.^[1]^ CDPX2, also known as Conradi Hünermann syndrome, is caused by the mutation of Xp11-encoded EBP (*EBP*), which is a Δ8-Δ7 sterol isomerase. Clinical CDPX2 cases are predominantly female, as CDPX2 is fatal in males. Apart from radiographic stippling at the metaphysis, female patients present short stature, short proximal limbs, transient congenital ichthyosis along the Blaschko line, patchy hair loss, cataracts, and craniofacial defects during infancy or childhood. Surviving CDPX2 males are extremely rare, and survival is attributed to germ cell mosaicism.^[2,3]^

In this article, we reported a female CDPX2 case caused by a heterozygous *EBP* mutation. Unlike most case reports, this patient presented a severe phenotype, including severely asymmetric short limbs (as observed by ultrasound) by gestational week 24+5. Whole exome analysis of aborted tissue confirmed *EBP* mutation. The severe phenotype was considered to be related to X-chromosome inactivation.

## 2 Case report

**Figure 1:**
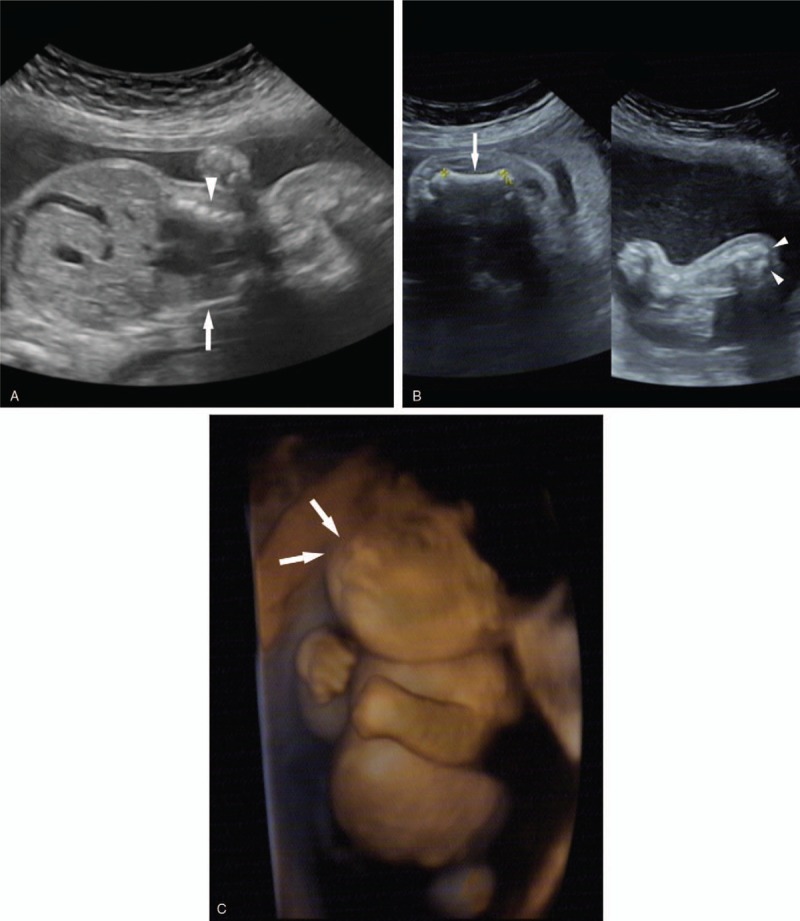
Ultrasound findings: (A) at gestational week 24+5: small and narrow thorax (arrowhead), chest circumference 15.6 cm, abdominal circumference 21.1 cm, shorter spine (arrow); (B) at gestational week 24+5: bending and short femur (arrows), thick metaphysis with high echoes (arrowheads); and (C) flat face and nose bridge (arrows).

The patient's mother was 36 years old, with a total of 5 pregnancies and 1 live birth. Eleven years ago, she gave birth to a healthy boy via cesarean section. She previously had 2 artificial abortions and 1 spontaneous abortion, with no family history of hereditary diseases. Ultrasound test result was normal at gestational week 6. At gestational week 12, nuchal translucency of 1.3 mm was observed, with no positive findings. Non-invasive DNA analysis showed low trisomy 21 risk, low trisomy 18 risk, and low trisomy 13 risk. At gestational week 24+5, ultrasound examination showed abnormal development of the long bones of the limbs (the length of the long bones was shorter than 1% controlled to the same gestational week of normal fetus), thick metaphysis in the right lower limb, irregular vertebral arrangement, and a narrow and small thorax (Fig. 1 A–D). The patient's parents decided to terminate the pregnancy at 27 weeks of gestation considering this as a lethal skeletal dysplasia.

**Figure 2:**
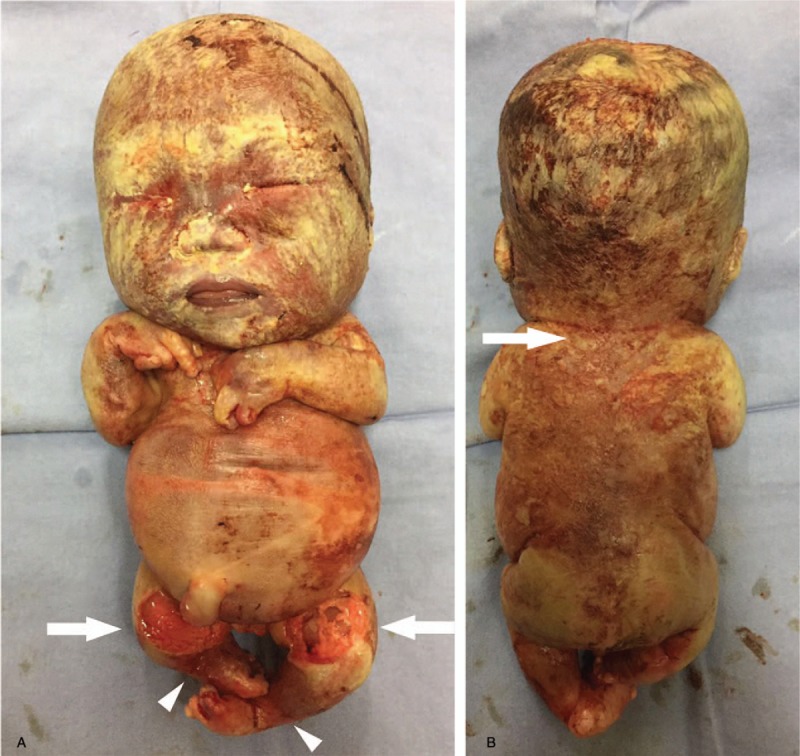
Gross images after delivery: (A) front: flat face, bilateral ankle joint contracture (arrowheads), asymmetric short lower limbs (arrows) and (B) back: short limbs, scale-like skin changes (arrow).

**Figure 3:**
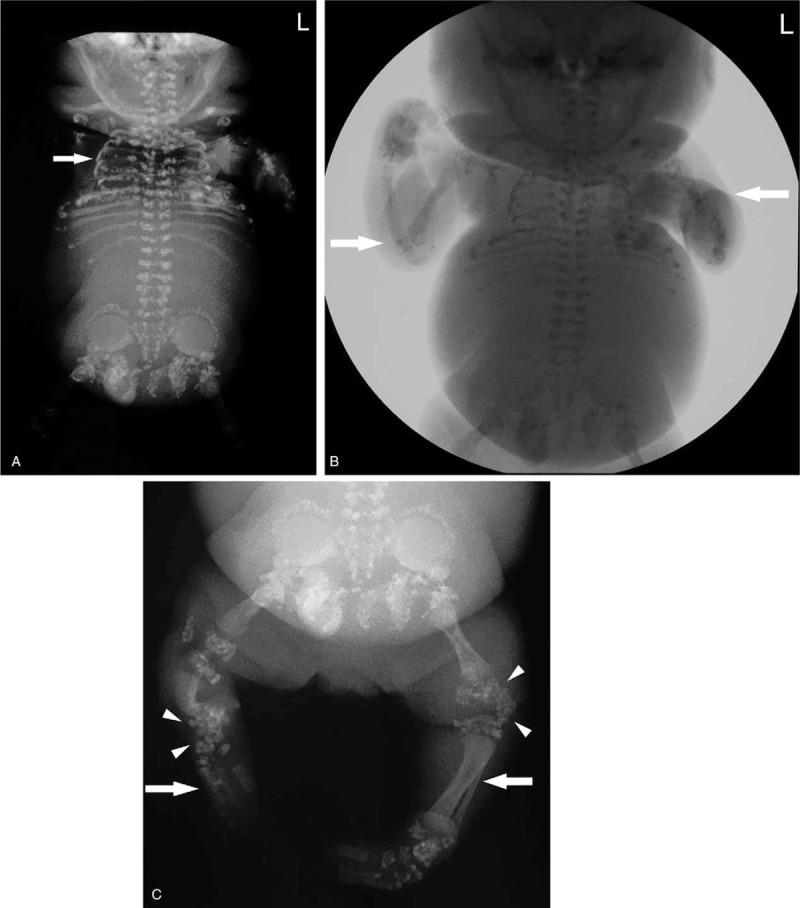
Post-delivery X-ray: (A) small and narrow thorax (arrows); (B) short limbs (arrows); and (C) asymmetric lower limbs (arrows), punctate changes in the metaphysic (arrowheads).

After the termination of pregnancy at week 27, the gross examination of the fetus showed a flat face and nose bridge, short limbs, asymmetric short lower limbs, and bilateral clubfeet with bilateral ankle joint contracture (Fig. 2 A, B). The autopsy report indicated that the left humerus bone was 2.5 cm long; the right humerus bone, 2.0 cm; the left femur, 3.0 cm; and the right femur, 2.2 cm; all were significantly shorter than 4SD measured at the same gestational age. In addition, the shapes of T3 to L3 were abnormal, the vertebral bodies appeared fused, the thorax was small with a maximum circumference of 16 cm, and the abdominal circumference was 26 cm. The autopsy also found subcutaneous edema in the head and face and thick metaphysis in the lower limbs. X-ray imaging showed short femur and humerus bones, a narrow and small thorax, thick metaphysis with a thick “splashed paint”’ pattern, and asymmetric short lower limbs (Fig. 3A–C).

**Figure 4:**
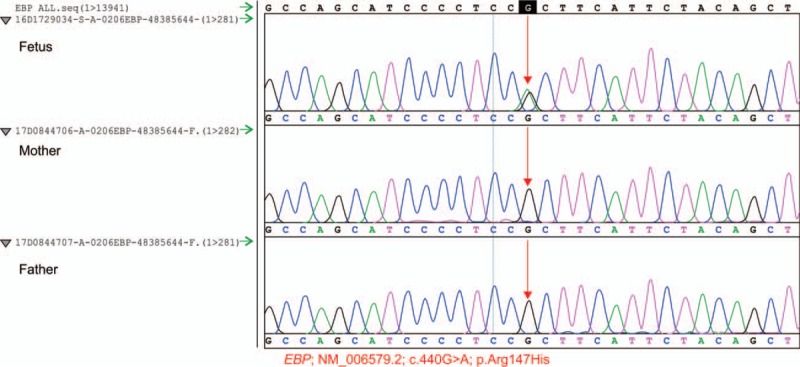
EBP whole exome analysis of the aborted tissue indicated a heterozygous EBP mutation, a known causative mutation; c.440G>A [p.Arg147His]) in the priori (fetus). A Sanger test showed that the patient's parents are non-carriers, indicating that the mutation is de novo. EBP = emopamil binding protein.

The karyotype of the fetus was 46, XX. No unusual single-nucleotide polymorphisms was detected. Whole exome analysis showed that the fetus was heterozygous for the *EBP* mutation (NM_006579.2; C.440G>A p.Arg147His), which must have occurred de novo because the parents were non-carriers (Fig. 4). Thus, CDPX2 was confirmed.

## 3 Discussion

The CDPX2 (OMIM 302960) is a rare biosynthesis disorder caused by an abnormality of post-squalene cholesterol due to *EBP* mutation. *EBP* is located on the short arm of the X chromosome (Xp11.22-p11.23) and spans 7 kb of genomic DNA. It has 5 exons that encode a 1-kb spliced transcript. EBP is widely expressed in all tissues and is located in the endoplasmic reticulum. It contains 230 amino acids, including 4 transmembrane domains, with a molecular weight of 27 kDa. To date, more than 70 *EBP* mutants have been identified. *EBP* encodes 3-beta hydroxysteroid Δ8-Δ7 isomerase, which catalyzes the isomerization of cholest-8(9)-ene-3β-ol to ketene sterols, thereby converting lanosterol to cholesterol; thus, Δ8-Δ7 isomerase is a key enzyme in the final step of sterol synthesis.^[4]^*EBP* mutation causes 8-dehydrocholesterol (8-DHC) and 8(9)-cholesterol to accumulate in the plasma, skin, and other tissues, resulting in extensive abnormalities.

Herman et al studied mutations in 26 female patients with suspected CDPX2. Among these 26 patients, 22 had *EBP* mutations, of which 13 mutations were de novo.^[5]^ For CDPX2, most EBP mutations are single-nucleotide changes (nonsense and missense) in exons. Although most mutations occur in exons, splice site mutations have also been reported. Arg147His, Trp61X, and Arg110Gln are frequently reported mutation sites, but the causes of these repeated hotspot mutations are unknown.^[6–10]^ Ikegawa et al concluded that truncation of the protein due to nonsense *EBP* mutation results in canonical CDPX2, and missense mutation results in noncanonical CDPX2.^[11]^ More researchers believe that there is no correlation between the genotype and phenotype of CDPX2.^[8,9,12]^ In this report, a heterozygous *EBP* mutation (a known causative mutation; c.440G>A [p.Arg147His]) was the cause of CDPX2. This is a missense mutation (arginine to histidine). A Sanger test showed that the patient's parents were noncarriers, indicating that the mutation of the fetus occurred de novo. Whittock et al, Has et al, and Cañueto et al have previously reported cases related to this mutation site.^[7–9]^

Because *EBP* is located on the X chromosome, mutations are usually fatal in male fetuses. Female fetuses that are heterozygous for the mutation usually present mild signs and symptoms: they have skin lesions and punctate calcifications in the long bones that gradually improve over time. Because the clinical symptoms are mild, female CDPX2 carriers are sometimes difficult to identify. In familial cases, the mother sometimes presents few clinical symptoms or presents milder clinical symptoms than her daughter.^[10]^

In this report, a 27-week-old female fetus presented markedly short bones, ankle joint contracture, markedly asymmetric short lower limbs, and a flat face and nose bridge. The severity of the phenotype was considered to be related to X-chromosome inactivation, also known as lyonization. For CDPX2, random X-chromosome inactivation during embryogenesis means that some cells carry a mutated *EBP* copy, whereas other cells carry a wild-type copy and produce normal cells. This phenomenon also explains the asymmetry of skeletal abnormalities. Several processes may occur to interfere with the randomness of X-chromosome inactivation, leading to a dominant maternal or paternal copy, also known as skewed X inactivation. If the wild-type X chromosome is inactivated in a high percentage of cells, skewed X inactivation may have a significant effect on the severity of X-linked disease in heterozygous females.^[9,10,13]^ Shirahama et al evaluated the potential effects of methylation and skewed X inactivation in CDPX2.^[10]^ Two patients with X-chromosome inactivation presented severe phenotypes, whereas their mothers presented mild phenotypes. This finding suggested that in some familial cases, X-chromosome inactivation may be the basis of CDPX2, especially when more severe clinical phenotypes occur in heterozygous females. Lefebvre, a French researcher, investigated the relationship between the genotypes and phenotypes of 9 female CDPX2 patients and concluded that the randomness of X-chromosome inactivation in various tissues may be related to the severity of female cases.^[14]^

*EBP* mutations lead to isomerase deficiency and accumulation of 8(9)-cholesterol and 8-DHC. Therefore, some researchers believe that measuring the levels of certain sterols in the plasma, amniotic fluid, and tissues may facilitate the diagnosis of CDPX2. The serum levels of sterols are highly specific and sensitive indicators of *EBP* mutations in suspected female CDPX2 cases.^[5]^ However, sometimes the levels of sterols may be misleading. Further screening of *EBP* mutations is recommended for highly suspected CDPX2 cases with normal sterol levels.

Some researchers have investigated the pathophysiological basis of severe deformities caused by defects in post-squalene cholesterol synthesis. Sterols are known precursors of biologically active compounds. For example, cholesterol is a precursor of bile acids, oxysterols, and steroids, and 7-dehydrocholesterol is a precursor of vitamin D3. It is now known that cholesterol is a ubiquitous component of all membranes and plays a special role in stabilizing lipid rafts. Thus, cholesterol plays a key role in establishing skin barrier permeability, and abnormalities in distal cholesterol synthesis may cause skin lesions in CDPX2.^[15]^ Moreover, cellular cholesterol is essential for cell growth and proliferation. Cholesterol plays a role in the autocleavage and activation of the hedgehog proteins. It directly affects the proteins involved in cell cycle regulation and affects organogenesis through cytoskeletal reorganization. The accumulation of toxic sterol intermediates from abnormal sterol metabolism may interfere with cholesterol-mediated protein modification, leading to bone malformations in CDPX2.^[16]^

CDPX2 must be differentiated from other short limb deformities, including skeletal dysplasia, other genetic or metabolic diseases, and vitamin K metabolic diseases (e.g., fetal exposure to the anticoagulant warfarin, or vitamin K epoxide reductase deficiency). Other diseases that may present punctate metaphysis include Zellweger (brain–liver–kidney) syndrome and metabolic diseases such as Smidth–Lemli–Opitz syndrome and GM1 gangliosidosis.

## 4 Conclusions

The CDPX2 is a type of skeletal dysplasia caused by *EBP* mutations. Female patients usually present a mild phenotype, but CDPX2 is fatal in male patients. It is still difficult to diagnose CDP by using ultrasound before delivery. CDP should be considered if prenatal ultrasound shows high punctate echoes at the metaphysis of long bones and asymmetrical short lower limbs. CDP can be confirmed with postnatal X-rays and genetic testing. The diagnostic value of sterol levels in the amniotic fluid and tissues has yet to be established. Fetal genotype and phenotype may be discordant, especially in female fetuses. Female cases with severe phenotypes may be associated with random inactivation of the X chromosome, also known as lyonization.

## Acknowledgments

The authors thank the family for participation in the present study.

## Author contributions

Yan Liu, first author, case analysis, writing the article; Qingqing Wu, critical revision of the article and final approval of article; Li Wang, prenatal ultrasound scanning; Bin Xu, postnatal X-ray imaging; Yike Yang, collection of case materials; Dan Shan, critical revision of article.

**Conceptualization:** Qingqing Wu.

**Data curation:** Yan Liu, Yike Yang.

**Investigation:** Yan Liu.

**Methodology:** Yike Yang.

**Resources:** Li Wang.

**Supervision:** Dan Shan.

**Visualization:** Li Wang, Bin Xu.

**Writing – original draft:** Yan Liu.

**Writing – review & editing:** Qingqing Wu.

Yan Liu orcid: 0000-0003-1698-5783.

## References

[1] Chen H Atlas of Genetic Diagnosis and Counseling[M]. 2012;New York: Springer, 345-352.

[2] Arnold AW Bruckner-Tuderman L Has C Conradi-Hunermann-Happle syndrome in males vs. MEND syndrome (male EBP disorder with neurological defects). Br J Dermatol 2012;166:1309–13. 22229330 10.1111/j.1365-2133.2012.10808.x

[3] Aughton DJ Kelley RI Metzenberg A X-linked dominant chondrodysplasia punctata (CDPX2) caused by single gene mosaicism in a male. Am J Med Genet A 2003;116a:255–60. 12503102 10.1002/ajmg.a.10852

[4] Herman GE Disorders of cholesterol biosynthesis: prototypic metabolic malformation syndromes. Hum Mol Genet 2003;12:R75–88. 12668600 10.1093/hmg/ddg072

[5] Herman GE Kelley RI Pureza V Characterization of mutations in 22 females with X-linked dominant chondrodysplasia punctata (Happle syndrome). Genet Med 2002;4:434–8. 12509714 10.1097/00125817-200211000-00006

[6] Braverman N Lin P Moebius FF Mutations in the gene encoding 3 beta-hydroxysteroid-delta 8, delta 7-isomerase cause X-linked dominant Conradi-Hunermann syndrome. Nat Genet 1999;22:291–4. 10391219 10.1038/10357

[7] Has C Bruckner-Tuderman L Muller D The Conradi-Hunermann-Happle syndrome (CDPX2) and emopamil binding protein: novel mutations, and somatic and gonadal mosaicism. Hum Mol Genet 2000;9:1951–5. 10942423 10.1093/hmg/9.13.1951

[8] Whittock NV Izatt L Mann A Novel mutations in X-linked dominant chondrodysplasia punctata (CDPX2). J Invest Dermatol 2003;121:939–42. 14632217 10.1046/j.1523-1747.2003.12489.x

[9] Canueto J Giros M Ciria S Clinical, molecular and biochemical characterization of nine Spanish families with Conradi-Hunermann-Happle syndrome: new insights into X-linked dominant chondrodysplasia punctata with a comprehensive review of the literature. Br J Dermatol 2012;166:830–8. 22121851 10.1111/j.1365-2133.2011.10756.x

[10] Shirahama S Miyahara A Kitoh H Skewed X-chromosome inactivation causes intra-familial phenotypic variation of an EBP mutation in a family with X-linked dominant chondrodysplasia punctata. Hum Genet 2003;112:78–83. 12483303 10.1007/s00439-002-0844-x

[11] Ikegawa S Ohashi H Ogata T Novel and recurrent EBP mutations in X-linked dominant chondrodysplasia punctata. Am J Med Genet 2000;94:300–5. 11038443 10.1002/1096-8628(20001002)94:4<300::aid-ajmg7>3.0.co;2-3

[12] Has C Seedorf U Kannenberg F Gas chromatography-mass spectrometry and molecular genetic studies in families with the Conradi-Hunermann-Happle syndrome. J Invest Dermatol 2002;118:851–8. 11982764 10.1046/j.1523-1747.2002.01761.x

[13] Migeon BR The role of X inactivation and cellular mosaicism in women's health and sex-specific diseases. J Am Med Assoc 2006;295:1428–33. 10.1001/jama.295.12.1428 16551715

[14] Lefebvre M Dufernez F Bruel AL Severe X-linked chondrodysplasia punctata in nine new female fetuses. Prenat Diagn 2015;35:675–84. 25754886 10.1002/pd.4591

[15] Mirza R Hayasaka S Takagishi Y DHCR24 gene knockout mice demonstrate lethal dermopathy with differentiation and maturation defects in the epidermis. J Invest Dermatol 2006;126:638–47. 16410790 10.1038/sj.jid.5700111

[16] Porter JA Young KE Beachy PA Cholesterol modification of hedgehog signaling proteins in animal development. Science 1996;274:255–9. 8824192 10.1126/science.274.5285.255

